# Effect of omega-3 fatty acids supplementation on cardio-metabolic and oxidative stress parameters in patients with chronic kidney disease: a systematic review and meta-analysis

**DOI:** 10.1186/s12882-021-02351-9

**Published:** 2021-05-01

**Authors:** Siavash Fazelian, Fatemeh Moradi, Shahram Agah, Akramsadat Hoseini, Hafez Heydari, Mojgan Morvaridzadeh, Amirhosein Omidi, Ana Beatriz Pizarro, Atie Ghafouri, Javad Heshmati

**Affiliations:** 1 Clinical Research Development Unit, Ayatollah Kashani Hospital, Shahrekord University of Medical Sciences, Shahrekord, Iran; 2 Department of Community Nutrition, School of Nutrition and Food Science, Isfahan University of Medical Sciences, Isfahan, Iran; 3 Colorectal Research Center, Iran University of Medical Sciences, Tehran, Iran; 4 Department of Education and Health Promotion,School of Health, Iran University of Medical Sciences, Tehran, Iran; 5 Cellular and Molecular Research Center, Sabzevar University of Medical Sciences, Sabzevar, Iran; 6 Department of Nutritional Science, School of Nutritional Science and Food Technology, Kermanshah University of Medical Sciences, Farabi Hospital, Faculty of Nutrition Sciences and Food Technology, Postal Code: 6715847141, Isar Square, Kermanshah, Iran; 7 Pontificia Universidad Javeriana, Bogotá, Colombia; 8 Department of Nutrition, School of Public Health, Iran University of Medical Sciences, Tehran, Iran

**Keywords:** Cardio-metabolic outcomes, CKD, Omega-3 FAs, Oxidative stress, Blood lipids

## Abstract

**Background:**

*Omega-3 fatty acids (*FAs) *have been suggested as a beneficial supplement in chronic kidney disease (CKD) patients, but the results of randomized clinical trials (RCTs) are controversial. We conducted a systematic review and meta-analysis to evaluate all the RCTs about the impact of* omega-3 FAs supplementation on cardiometabolic outcomes and oxidative stress parameters in patients with CKD.

**Methods:**

We performed a systematic database search in *PubMed/MEDLINE, EMBASE, Scopus, Web of Science, and Cochrane Central, up to May 2020.* We included all placebo-controlled randomized trials that assessed the effect of *omega-3 FAs supplementation on* any *cardiometabolic outcomes*: blood pressure, total cholesterol (TC), low-density lipoprotein (LDL) and high-density lipoprotein (HDL) or triglycerides (TG) and oxidative stress parameters. Data were pooled using DerSimonian–Laird’s random-effects model.

**Results:**

Finally, thirteen articles met the inclusion criteria for this review omega-3 FAs supplementation significantly decrease TC (SMD: -0.26; 95% CI: − 0.51, − 0.02; I^2^ = 52.7%), TG (SMD: -0.22; 95% CI: − 0.43, − 0.02; I^2^ = 36.0%) and Malondialdehyde (MDA) levels (SMD: -0.91; 95% CI: − 1.29, − 0.54; I^2^ = 00.0%) and also significantly increase superoxide dismutase (SOD) (SMD: 0.58; 95% CI: 0.27, 0.90; I^2^ = 00.0%) and Glutathione peroxidase (GPx) (SMD: 0.50; 95% CI: 0.14, 0.86; I^2^ = 00.0%) activities. However our results show that omega-3 FAs supplementation have no significant effects on HDL, LDL and blood pressure.

Conclusion

This systematic review and meta-analysis supports current evidence for the clinical benefit of omega-3 FAs intake to improve cardiometabolic parameters in CKD patients. However, well-designed RCTs still needed to provide a conclusive picture in this field.

**Supplementary Information:**

The online version contains supplementary material available at 10.1186/s12882-021-02351-9.

## Highlights

- *Chronic kidney disease (CKD) is the progressive loss of renal function that recognized as a global public health problem*
- *Omega-3 fatty acids (*FAs) *have beneficial supplement in chronic kidney disease (CKD) patientssss*
- Omega-3 FAs intake significantly improves lipid profile and increases antioxidant defense enzymes in CKD patients.

## Background

Long-chain Omega-3 fatty acids such as, docosahexaenoic acid (DHA) and eicosapentaenoic acid (EPA), are polyunsaturated fatty acids that mostly intake from seafood [1, 2]. Omega-3 fatty acids (FAs) are widely used to manage disease and complications [3]. Omega-3 FAs because of antioxidant properties can protect the cardiovascular system from oxidative stress and thrombotic complications [4].

Patients with chronic kidney diseases (CKD) who are maintained with dialysis or nephropathy have a risk of coronary heart disease, myocardial infarction, and atherosclerosis [5, 6]. Cardiometabolic factors such as hypertension, dyslipidemia, hyperglycemia, and obesity are determinant risk factors in the general population [7, 8]. Cardiometabolic risk factors impose a great burden on health and insurance systems all over the world [9, 10] and they are even more harmful in people with underlying diseases such as CKD [11–13]. Elevated levels of low-density lipoprotein (LDL), accumulate remnant lipoproteins as well as oxidized LDL (ox-LDL) and generate atherosclerosis [14]. Patients, undergoing hemodialysis, are at higher risk due to metabolic changes, increased lipid profile can result in unpleasant disorders that must be prevented [15]. However, lipid profile especially TC levels considered as nutritional parameters [16], which have been demonstrated to be strictly related to cardiometabolic risks in CKD patients [17].

Patients with end-stage renal disease (ESRD) do not receive enough omega-3 fatty acids due to limited consumption of rich food of omega-3 fatty acids, such as nuts and fishes [18]. Some of the RCTs indicated the administration of n-3 fatty acids has a beneficial effect on serum lipid profile, but maybe depending on the selected dose [19]. Recent randomized controlled trials (RCTs) show omega-3 supplements (EPA and DHA) usually have opposite effects versus placebo [18, 20].

Previous clinical trials have examined the effect of omega-3 supplements on cardiometabolic factors that have been performed in patients with CKD, but no meta-analysis and systematic review reported the overall impact of n-3 PUFA clinical trials. Thus, our study aimed to evaluate the overall effects of oral omega-3 supplementation on serum lipid profile, hypertension and antioxidant markers in CKD patients.

## Methods

### Search strategy

This meta-analysis was performed according to the PRISMA guidelines [21]. Electronic databases, including PubMed/MEDLINE, EMBASE, Scopus, ISI Web of Science, and Cochrane Central, were systematically searched up to May 2020. The search strategy was conducted without any language or time restriction. Mesh terms were used to PubMed and topic heading titles were used for other databases search. The systematic search was completed using the following terms: Fish Oil OR Fatty Acids, Omega-3 OR Docosahexaenoic Acids OR “DHA” OR Eicosapentaenoic Acid OR “EPA” OR Timnodonic Acid AND Dialysis OR Hemodialysis OR Peritoneal dialysis OR Kidney disease OR Chronic kidney disease OR End stage renal disease OR Chronic renal failure AND Randomized controlled trial OR controlled clinical trial OR randomized controlled trials. Complete search terms and syntaxes are presented in Additional file 1.

### Study selection & quality appraisal

The titles and abstracts of the studies were screened by two independent investigators (S.F and F.M), then the full text of the related articles was checked, and any disagreements were resolved by consensus. All human clinical trials that reported the effect of omega-3 supplementation on cardiometabolic factors in chronic kidney patients were included in the current meta-analysis. Experimental studies, trials that used a combination of omega-3 and other interventions and studies that used olive oil or medium-chain triglyceride (MCT) as a placebo, were excluded. The primary outcomes of this review were cardiometabolic factors such as blood lipids (TC, TG, LDL and HDL), and systolic or diastolic blood pressure. The secondary outcomes were oxidative stress parameters (TAC, MDA, SOD and Gpx activity). We evaluated the methodological quality of each included trial using criteria from the Cochrane Collaboration guidelines [22].

### Data extraction and data analysis

The review of primary studies was performed by one author (S.F) and then the data were double-checked by a second one (F.M) and any discrepancies were discussed by the other author (J.H), we extracted general characteristics of the study include of first author’s name, year of publication, country, subjects, sample size, mean age, gender, dose and type of supplemented omega-3 and main outcomes that has been indicated in Table 1. The mean and standard deviation of the primary outcomes associated with cardiometabolic were extracted from related articles for continuous and binary data we calculated the standardized mean difference (SMD). Standard Errors (SE), confidence interval, *interquartile range* (*IQR*), and minimum-maximum value of each cardiometabolic marker in omega-3 and placebo group were converted to Standard Deviations (SD).

**Table 1:** Main characteristics of included studies

Study (year)	Country	Subjects	Sample size	N-3 fatty acids Dosage (per day)				Duration (week)	Gender (M/F)	Age		Main outcome
				N-3 Dose (mg)	EPA (mg)	DHA (mg)	ALA (mg)			Placebo Mean ± SD	Intervention Mean ± SD	
**Ando** **et al** **[**23**]** **(1999)**	**Japan**	HD ^a^	38	1800	1638	–	–	12	33/5	51 ± 13	54 ± 11	↓TC ^c^, ↓TG ^d^, ↔HDL ^e^
**Ateya** **et al** **[**24**]** **(2017)**	**Egypt**	HD	49	1000	500	250	–	16	27/22	14.6 ± 2.7	14.7 ± 2.7	↔TC, ↓TG, ↔HDL, ↔LDL ^f^, ↔MDA ^g^ ,↑GPx ^h^,↑SOD ^i^
**Bouzidi** **et al** **[**25**]** **(2010)**	**Algeria**	CRF ^b^	40	2100	693	252	–	12	22/18	61 ± 14	61 ± 14	↓TG, ↑GPx, ↑SOD, ↔HDL, ↔LDL, ↔TC
**Alexopoulos** **et al** **[**26**]** **(2004)**	**Greece**	IgA Nephropathy	28	3000	850	580		192	22/6	39 ± 10	41 ± 12	↔TG
**Gharekhani** **et al** **[**27**]** **(2016)**	**Iran**	HD	45	1800	1080	720	–	16	25/20	57.2 ± 15.19	56.8 ± 13.09	↔LDL,↓TG,↓TC,↓HDL
**Jabbari** **et al** **[**28**]** **(2016)**	**Iran**	HD	117	3000		–		12	75/25	61.05 ± 17.42	64.58 ± 12.61	↔TC, ↔TG, ↔LDL , ↑HDL
**Khajehdehi** **[**29**]** **(2000)**	**Iran**	HD	30	1500		–		8	15/15	32.4 ± 9.2	32.7 ± 10.7	↔TC, ↓ TG, ↓LDL, ↑HDL
**Moeinzadeh** **[**30**]** **et al (2011)**	**Iran**	HD	52	3000	540	360	–	24	36/16	58.34 ± 14.36	57.76 ± 15.56	↑HDL, ↔LDL, ↔TC, ↔TG
**Naini et al** **[**31**]** **(2015)**	**Iran**	PD^l^	90	3000	540	360	–	8	51/39	59.36 ± 13.4	57.7 ± 16.3	↓DBP ^j^, ↓SBP ^k^, ↔HDL ,↔LDL, ↔TC, ↔TG
**Pettersson** **[**32**]** **et al** **(1994)**	**Sweden**	IgA Nephropathy	32	6000	2800	1530	–	24	25/7	42	39	↔HDL, ↔TC ,↓TG, ↔LDL, ↔SBP, ↔DBP
**Schmitz** **[**33**]** **et al** **(2002)**	**USA**	HD	24	4000	1760	960	–	48	11/13	54 ± 3	52 ± 6	↓TG
**Tayyebi-Khosroshahi et al** **[**34**]** **(2013)**	**Iran**	HD	88	3000	540	360	–	8	63/25	48.60	51.50	↔HDL, ↔LDL, ↔TC, ↔TG
**Tayyebi-Khosroshahi et al** **[**35**]** **(2010)**	**Iran**	HD	75	3000	540	360	–	8	49/26	49.3 ± 1.8	48.8 ± 2.7	↑GPX, ↓MDA, ↑SOD,

^a^ Hemodialysis, ^b^Chronic Renal Failure, ^c^ Total cholesterol, ^d^ Triglyceride, ^e^ *High-density lipoprotein,* ^f^ *low-density lipoprotein*, ^g^ Malondialdehyde*,* ^h^ *Glutathione* peroxidase*,* ^i^ *Superoxide dismutase,* ^j^ *diastolic blood pressure,* ^k^ *Systolic blood pressure,* ^l^ *peritoneal dialysis*

All statistical analysis was performed with a random effect model based on the Inverse-Variance method using STATA software version 13 (STATA Corp, College Station, Texas). We assessed the heterogeneity of studies using heterogeneity chi-squared tests with a *P*-value of less than 0.1 and I^2^ statistic more than 50% considered as significant heterogeneity [22]. Subgroup analysis based on duration and dose of omega-3 FAs supplementation was conducted. The funnel plot, Begg and Egger test were used to evaluate the publication bias of included trials [36]. Sensitivity analysis was performed to assess the extent to which inferences might depend on an individual trial.

## Results

### Search results

**Fig. 1:**
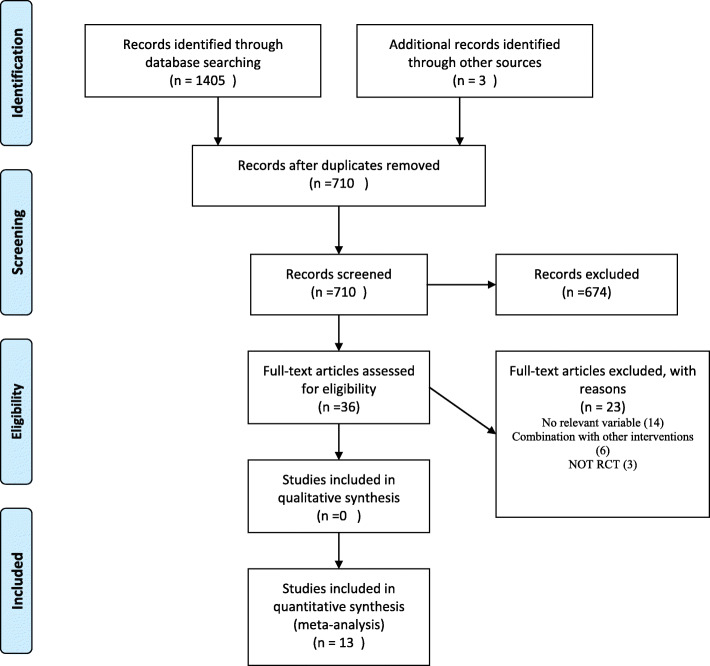
PRISMA Flow diagram of study selection

The study identification and study selection process is presented in the PRISMA flow diagram in Fig. 1. Initial database search yielded an aggregate of 1405 potentially relevant records. After reading the title and abstract of duplicate removed records, 36 studies remained for the comprehensive full-text evaluations. Of these 36 articles, 23 more studies were excluded for various reasons. Finally, 13 articles [23–35] met the inclusion criteria for this review.

### Study characteristics

The main characteristics of the included articles are presented in Table 1. All the included studies were published between 1994 and 2017. The included trials originated from the Iran (*n* = 7), the United States (*n* = 1), Egypt (n = 1), Algeria (n = 1), Japan (n = 1), Sweden (n = 1), and Greece (n = 1). The total number of participants in all included trials was 708. Ten studies evaluated the effect of omega-3 FAs on hemodialysis patients [23, 24, 27–31, 33–35], two IgA nephropathy patients [26, 32] and one in chronic renal failure patients [25]. The duration of omega-3 supplementation in studies ranged from 8 to 192 weeks. The mean age of participants in included studies ranged from 14.1 to 64 years old. Among the 13 included studies, one used EPA-only supplements [23], and the other 12 trials involved supplementation of EPA/DHA combinations. Omega-3 FAs dosage ranged from 1000 to 6000 mg/day in trials.

### Risk of bias assessment

The risk of bias evaluation of included studies is shown in Additional file 2. Overall, seven trials reported on random sequence generation [26–28, 30, 31, 33, 34], seven reported precise and correct allocation concealment [23, 24, 27, 30, 31, 33, 34]. Five trials were evaluated as having a high risk of performance bias [23, 26, 28, 29, 35] and only two studies have evaluated as have a low detection bias [25, 31]. Three studies were considered to have attrition bias [25, 30, 32] and the risk of selective reporting was high in two trials [23, 32].

### Blood pressure

**Fig. 2:**
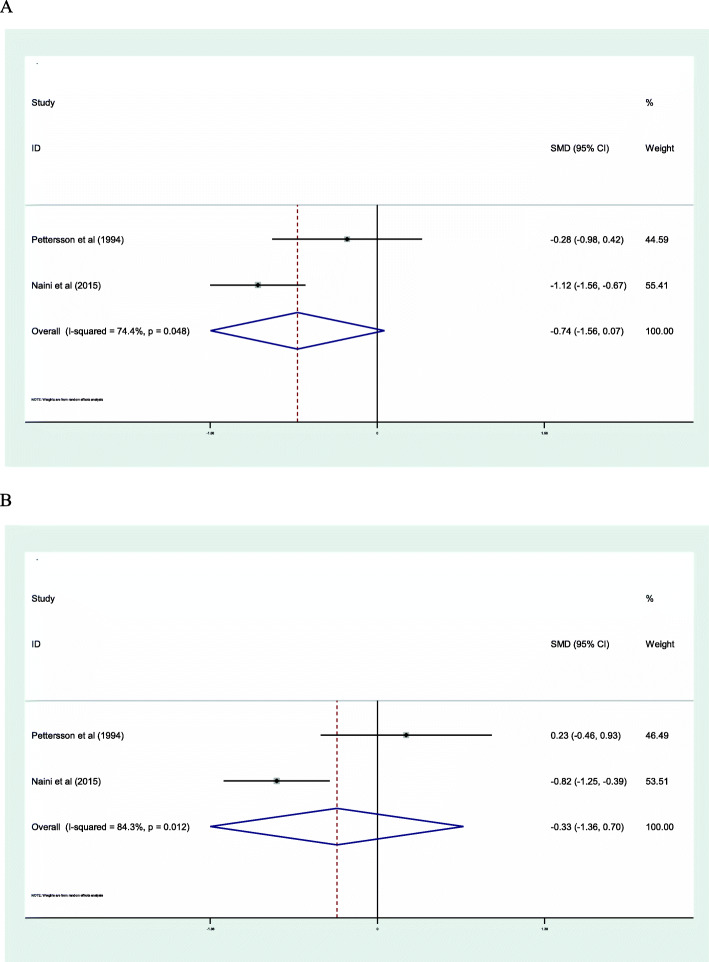
Forest plot of the effect of omega-3 FAs supplementation on SBP (**a**) and DBP (**b**) in CKD patients

Two included studies evaluated the effects of omega-3 FAs supplementation on blood pressure in CKD patients. The results of meta-analysis show that omega-3 FAs supplementation have no effect on SBP (SMD: -0.74; 95% CI: − 1.56, 0.07; I^2^ = 74.4%) (Fig. 2**a**) and DBP (SMD: -0.33; 95% CI: − 1.36, 0.70; I^2^ = 84.3%) (Fig. 2**b**).

### Blood lipids

**Fig. 3:**
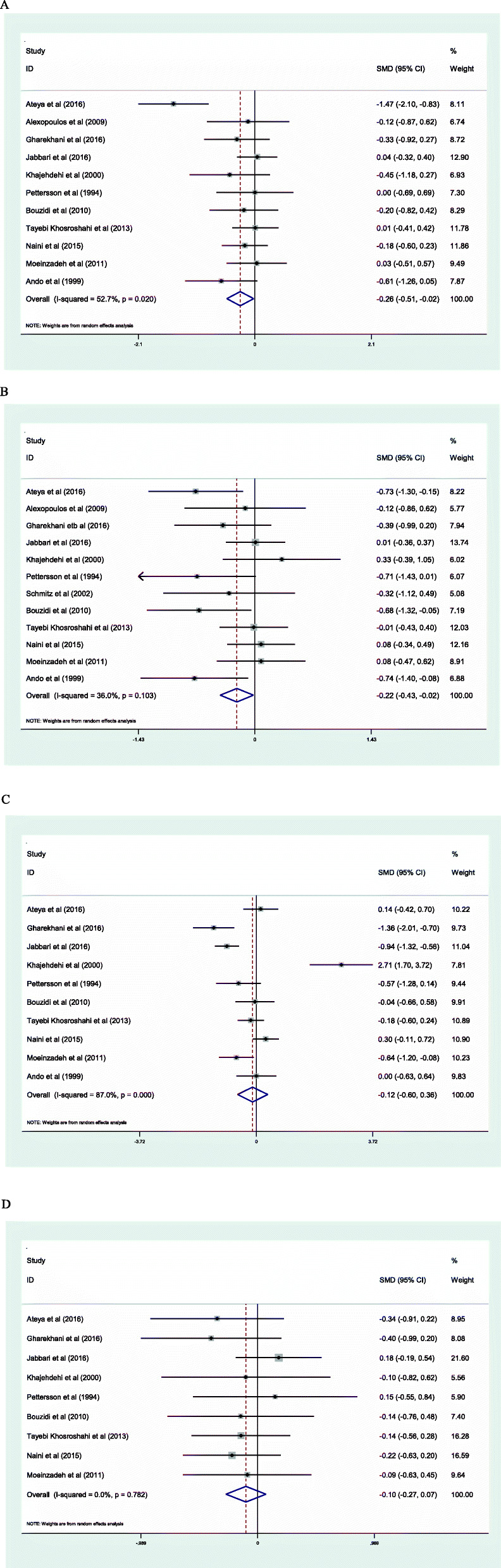
Forest plot of the effect of omega-3 FAs supplementation on TC (**a**) TG (**b**), HDL (**c**) and LDL (**d**) in CKD patients

Meta-analysis of 11 included trials show that omega-3 FAs supplementation significantly reduce TC levels in CKD patients (SMD: -0.26; 95% CI: − 0.51, − 0.02; I^2^ = 52.7%) (Fig. 3**a**). Subgroup analysis based on duration did not show any significant changes (Table. 2), but subgroup analysis based on the dose of omega-3 FAs supplementation shows that omega-3 FAs supplementation is less or equal to 2000 mg/day significantly decrease TC levels (Table. 2). Meta-analysis of 12 included studies demonstrated that omega-3 FAs supplementation significantly reduce TG levels in CKD patients (SMD: -0.22; 95% CI: − 0.43, − 0.02; I^2^ = 36.0%) (Fig. 3**b**). stratification of results according to the duration of omega-3 FAs supplementation indicates that TG decreased significantly in more than 10 weeks of omega-3 FAs supplementation (SMD: -0.36; 95% CI: − 0.63, − 0.10; I^2^ = 38.3%) (Table. 2). According to omega-3 FAs doses, subgroup analysis did not show any significant changes (Table. 2). Meta-analysis on ten studies that evaluated the effect of omega-3 FAs on HDL in CKD patients did not show significant changes (SMD: -0.12; 95% CI: − 0.60, 0.36; I^2^ = 87.0%) (Fig. 3**c**). stratification based on omega-3 FAs intake duration indicated that omega-3 FAs decreased HDL levels in more than 10 weeks of duration in CKD patients (SMD: -0.49; 95% CI: − 0.90, − 0.09; I^2^ = 71.8%) (Table. 2) compare to less and equal than 10 weeks (SMD: 0.83; 95% CI: − 0.32, 1.97; I^2^ = 92.6%). Subgroup analysis based on omega-3 FAs doses did not show any significant changes (Table. 2). Nine trials evaluated the effect of omega-3 FAs on LDL levels in CKD patients. Meta-analysis indicated that effect omega-3 FAs supplementation did not have significant effects on LDL levels (SMD: -0.10; 95% CI: − 0.27, 0.07; I^2^ = 00.0%) (Fig. 3**d**). Subgroup analysis based on duration and dose of omega-3 FAs supplementation did not show any significant changes in this effect (Table 2).

**Table 2:** Subgroup analysis assessing the effect of omega-3 FAs supplementation on metabolic parameters in CKD patients

Variable	Sub-grouped by		No. of arms	effect size (SMD)	95% CI	I^**2**^ (%)	P for heterogeneity
TC	Duration	> 10 weeks	8	−0.31	− 0.66, 0.03	64.1	**0.007**
		≤10 weeks	3	−0.14	− 0.41, 0.13	00.0	0.540
	omega-3 FAs dosage	≤ 2000 mg/day	5	**−0.61**	**−1.07, − 0.14**^a^	58.9	**0.045**
		> 2000 mg/day	6	−0.04	−0.23, − 0.15	00.0	0.961
TG	Duration	> 10 weeks	8	**−0.36**	**−0.63, − 0.10**^a^	38.3	0.125
		≤10 weeks	3	0.03	−0.23, 0.29	00.0	0.692
	omega-3 FAs dosage	≤ 2000 mg/day	5	−0.36	− 0.74, 0.02	40.5	0.151
		> 2000 mg/day	7	−0.12	−0.35, 0.10	23.7	0.249
HDL	Duration	> 10 weeks	7	**−0.49**	**−0.90, − 0.09**^a^	71.8	**0.002**
		≤10 weeks	3	0.83	−0.32, 1.97	92.6	**0.000**
	omega-3 FAs dosage	≤ 2000 mg/day	5	0.04	−0.97, 1.06	93.0	**0.000**
		> 2000 mg/day	5	−0.18	− 0.53, 0.17	55.2	0.063
LDL	Duration	> 10 weeks	6	−0.06	−0.27, 0.16	00.0	0.513
		≤10 weeks	3	−0.17	−0.44, 0.10	00.0	0.948
	omega-3 FAs dosage	≤ 2000 mg/day	4	−0.10	−0.40, 1.20	22.0	0.278
		> 2000 mg/day	5	−0.13	−0.35, 0.10	00.0	0.937

*SMD* Standard mean difference, *CI* confidence interval. *TC* Total cholesterol, *TG* Triglyceride, *HDL* High density lipoprotein, LDL Low density lipoprotein

^a^statistically significant

### Oxidative stress

**Fig. 4:**
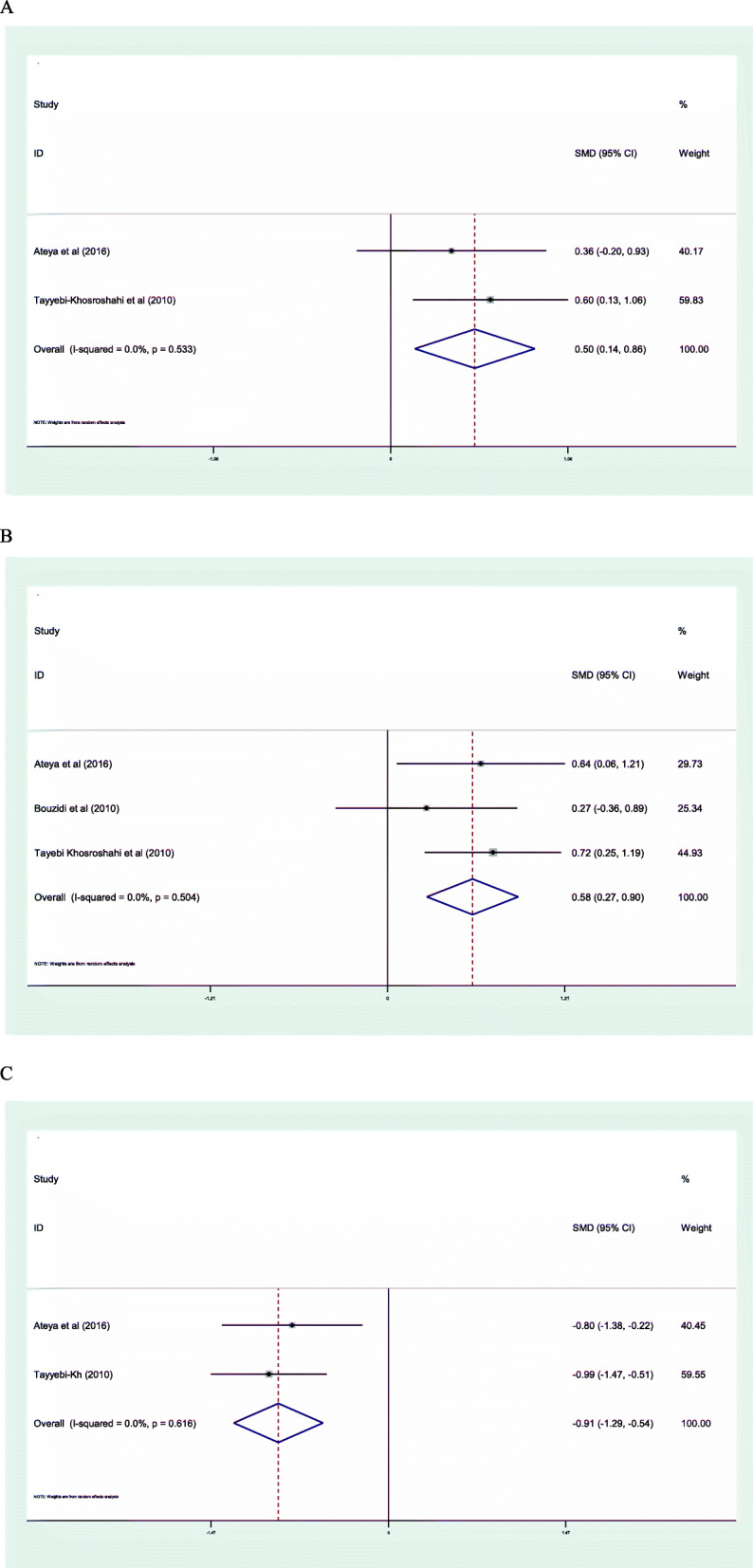
Forest plot of the effect of omega-3 FAs supplementation on oxidative stress parameters; GPx(**a**), SOD (**b**) and MDA (**c**)

The result of our meta-analysis indicated that omega-3 FAs supplementation significantly increase GPx (SMD: 0.50; 95% CI: 0.14, 0.86; I^2^ = 00.0%) (Fig. 4**a**), and SOD activities (SMD: 0.58; 95% CI: 0.27, 0.90; I^2^ = 00.0%) (Fig. 4**b**), and also significantly decrease MDA levels (SMD: -0.91; 95% CI: − 1.29, − 0.54; I^2^ = 00.0%) (Fig. 4**c**) in CKD patients.

### Sensitivity analysis and publication bias assessment

The main outcomes of the meta-analysis did not alter after drop out of any single of the included trials except for the condition as follow: The significant finding of the meta-analysis of the effect omega-3 FAs supplementation on TC altered to “insignificant” after drop out of the dataset of Ateya et al. [24] According to Begg and Egger test there was no publication bias in our results. Findings of the funnel plots are presented in Additional file 3.

## Discussion

This systematic review and meta-analysis investigated the efficacy of omega-3 FAs supplementation on cardiometabolic outcomes and oxidative stress parameters in patients with CKD. Our results indicated that omega-3 FAs supplementation significantly reduces TC, TG, and MDA levels and substantially increases SOD and GPx activity. However, our results show that omega-3 FAs intake has no significant effects on HDL, LDL and blood pressure. Several systematic reviews evaluated the effects of omega-3 FAs intake on CKD patients. It should be noted that CKD is a very heterogeneous disease. IgA nephropathy, CKD, and HD patients are collectively referred to as CKD patients, but the significance of improving lipids, blood pressure, and oxidative stress markers differ depending on the pathology, so due to great heterogeneity current results should be declared with caution.

In line with our results, Saglimbene et al. in 2020 [37] demonstrated that omega-3 FAs intake might decrease cardiovascular mortality in hemodialysis patients, but it is uncertain whether omega-3 FAs intake reduces the risk of mortality or ESKD in subjects with CKD. Wu et al. [38] also indicated that omega-3 FAs supplementation might improve inflammatory factors in dialysis patients. In previous systematic reviews, omega-3 FAs intake has beneficial effects on the Lipid profile of CKD patients [39, 40].

Our results indicated that omega-3 FAs intake did not have a significant impact on systolic and dyastolic blood pressure in patients with CKD. However, the small number of included studies that evaluated the effect of omega-3 FAs intake on blood pressure in CKD patients makes it difficult for us to find a possible significant effect. In addition, significant reduction was observed just in Naini et al. [31] which is conducted on PD patients, so maybe this effect is related to the specific pathophysiology of PD patients. There are several studies that demonstrated the beneficial effect of omega-3 FAs supplementation on improvement of blood pressure [41, 42], so maybe if there were more RCTs, we could find a significant effect of omega-3 FAs intake on blood pressure in CKD patients.

Our results indicated that omega-3 FAs intake has no effect on HDL levels in CKD patients. However, the subgroup analysis based on duration showed that omega-3 FAs intake more than ten weeks of duration significantly decrease HDL levels. This result is in contrast with Zho et al. [40] that shows that omega-3 FAs intake significantly increases HDL levels in dialysis patients. Several reasons could be proposed for these different results between present the review, and Zho et al. various included studies may cause the primary reason for these discrepancies in results. In this field, Eslick et al. [39] indicated that fish oil intake may slightly (non-significantly) increase HDL levels. Our results also showed that omega-3 FAs intake has no effect on LDL levels in CKD patients. Nevertheless, we observed a non-significant decrease in LDL levels, and maybe if there were more trials, we could find this reduction significant. Because previous reviews also endorse that omega-3 FAs supplementation can be useful in improving LDL levels [43, 44]. Our meta-analysis results indicated that omega-3 FAs intake significantly decreases TC levels in patients with CKD. This outcome is in accordance with Zhu et al. [40] results. Several mechanisms could be proposed for the effect of omega-3 FAs intake on TC levels. It has been shown that omega-3 FAs can reduce cholesterol levels via suppression of endogenous cholesterol synthesis [45]. Actually, it has been suggested that omega-3 FAs intake caused a significant decrease in the mRNA expression levels of 3-Hydroxy-3-Methyl-Glutaryl-Coenzyme A reductase (HMG-CoA reductase) which is the limited rate enzyme in cholesterol synthesis [46]. Recently it has been shown that cholesterol metabolism also is regulated by microRNA regulation [47]. microRNAs (miRNAs) are small non-coding RNAs that play important roles in posttranscriptional gene regulation [48, 49]. Several studies have been demonstrated that omega-3 fatty acid regulates cholesterol metabolism through modulation of different miRNAs [50]. So maybe the effect of omega-3 supplementation on cholesterol related to the regulation of miRNAs. In the present study, subgroup analysis based on omega-3 FAs dose indicated that omega-3 FAs intake less than 2000 mg/day is more efficient to reduce TC levels compare to more than 2000 mg/day.

Our results also indicated that omega-3 FAs intake significantly reduces TG levels. This result is following previous findings that demonstrate the beneficial effects of omega-3 FAs intake on TG levels [51–53]. Our results also show that omega-3 FAs intake reduces TG more efficiently in more than ten weeks of duration compare to less or equal to 10 weeks. Omega-3 FAs may represent their hypotriglyceridemic impacts by several possible mechanisms. It has been demonstrated that omega-3 FAs can affect the genetic or epigenetic factors and related regulatory proteins [54–57], for instance, omega-3 FAs may inhibiting hepatic lipogenesis via decreasing levels of sterol receptor element-binding protein–1c (SREBP-1c), the leading genetic switch controlling lipogenesis [58, 59]. Omega-3 FAs have been shown to inhibit SREBP-1c transcription through a diminished trans-activating capacity of Liver X receptor alpha (LXRα) [60]. Besides it has been suggested that omega-3 FAs intake upregulates fatty acids oxidation in the skeletal muscle and liver via activation of Peroxisome proliferator-activated receptor (PPARs) [61, 62], and increasing flux of glucose to glycogen via suppression of hepatocyte nuclear factor–4α (HNF-4α) [63, 64]. The net outcome is the change in the source of metabolic fuel from storage TG toward oxidation.

Our results show that omega-3 FAs intake improves the antioxidant defense system through increase GPx and SOD activity and decrease MDA concentrations. However the number of included studies that evaluated the effect of omega-3 FAs supplementation on oxidative stress parameters in CKD patients was very limited, so this result should be declared with caution. The antioxidant effect of omega-3 FAs supplementation in improving oxidative stress parameters has been demonstrated in previous systematic reviews [65–67]. Oxidative stress is a pivotal contributor to cardiometabolic risk factors in CKD patients [68]. Therefore, reduces cell toxicity related to increasing apoptosis and cell death through regulation of oxidative stress is very critical to cell survival [69]. Mitochondrial biogenesis is strictly related to oxidative stress and cell survival [70]. SOD activity also is related to mitochondrial biogenesis [71]. In this regard, it has been shown that omega-3 FAs intake regulates mitochondrial biogenesis through increase SOD activity [72]. In addition to the effect of omega-3 on oxidative stress, it has been shown that omega-3 fatty acids have a strenght immunomodulatory effect which can affect antioxidant enzymes and lipid peroxidation parameters such as GPx and MDA [73]. Eicosanoids generated from arachidonic acid have critical roles in the regulation of the immune system [74]. Omega-3 fatty acids such as EPA and DHA produced different eicosanoids; these may have different functions to arachidonic acid-derived ones especially in the brain [75] and kidneys [76]. Human immune cells generally have a great amount of arachidonic acid, but arachidonic acid, DHA, and EPA amount of cells can be changed via oral intake of those fatty acids. It has been demonstrated that omega-3 FAs eicosanoids are more resistant to lipid peroxidation and decrease MDA levels [77].

### Strengths and limitations

Our systematic review has evaluated the impacts of omega-3 FAs intake on CKD patients. Our database search was without language restrictions and very comprehensive. We also assessed the quality of the included trials. We investigated the impacts of different factors including properties of the participants and treatment on the effects of the supplements. The included trials are very varied in the type of omega-3 FAs, dose and duration of administration. Besides, there is limited reported data about the compliance of intervention in participants. Moreover, even with contacting the authors, we could not get enough information in some of the included studies. Given the limited number of trials in each subgroup analysis, we could not conduct a meta-regression analysis. In addition, the evaluation the risk of CVD disease in CKD patients in different stages may not a good way to conclude the effect of omega-3 FAs intervention on cardio-metabolic risk factors in CKD patients. It is well established that the pathogenesis of cardio-metabolic parameters is different in HD patients and non-dialysis CKD patients. Therefore, the impact of fatty acids intervention on hard outcomes is thought to vary by CKD stage and there are controversial results in the literature. For example, it has been shown that the higher the TC and TG levels are associated with, the higher the risk of developing coronary artery disease in CKD patients [78], but interventions to reduce blood lipids indicated that these treatments are effective for decrease total mortality and CVD events in early CKD stages, but have little benefit in dialysis patients [79] or even did not significantly improve CVD-related hard endpoints when limited to hemodialysis patients [80]. Moreover of thirteen included studies, ten of them were performed on HD patients so the majority of results may be related to HD patients. In addition, significant heterogeneity was encountered perhaps due to various settings, populations enrolled etc., calling for cautious interpretation of the results. Also, the effect in many variables such as blood pressure was evaluated by very few included trials and the effect may related to a specific disease condition like PD patients; thus, the evidence to support it is low. Finally, the vast majority of included trials originated from Iran; thus, extrapolation of these results to Western should be considered with caution.

## Conclusion

This systematic review and meta-analysis support current evidence for a clinical benefit of omega-3 FAs intake to improve cardiometabolic parameters in CKD patients. These results show that omega-3 FAs intake significantly decrease TC, TG and MDA levels and also considerably increases SOD and GPx activity. However well-designed large randomized clinical trials still needed to provide a conclusive picture of the effect of omega-3 FAs supplementation on oxidative stress and blood pressure parameters in patients with CKD.

## Supplementary Information

**Additional file 1.**

**Additional file 2.**

**Additional file 3.**

## Data Availability

Not applicable.

## Abbreviations

CKD: Chronic kidney disease
RCTs: Randomized clinical trials
LDL: Low-density lipoprotein
HDL: High-density lipoprotein
TG: Triglycerides
TC: Total cholesterol
MDA: Malondialdehyde
GPx: Glutathione peroxidase
SOD: Superoxide dismutase
SMD: Standard mean difference
CI: Confidence interval
FAs: Fatty acids
DHA: Docosahexaenoic acid
EPA: Eicosapentaenoic acid
ox-LDL: oxidized low-density lipoprotein
ESRD: End-stage renal disease
MCT: Medium-chain triglyceride
TAC: Total antioxidant capacity
IQR: Interquartile range
SE: Standard Errors
SD: Standard Deviations
SBP: Systolic blood pressure
DBP: Diastolic blood pressure
HMG-CoA reductase: Hydroxy-Methyl-Glutaryl-Coenzyme A reductase
SREBP-1c: Sterol receptor element-binding protein–1c
LXRα: Liver X receptor alpha
PPARs: Peroxisome proliferator-activated receptor
HNF-4α: Hepatocyte nuclear factor–4α
