# Supplementary material for: Effect of omega-3 fatty acids supplementation on cardio-metabolic and oxidative stress parameters in patients with chronic kidney disease: a systematic review and meta-analysis
Source: BMC Nephrol. 2021 May 1;22:160. doi: 10.1186/s12882-021-02351-9 (PMC8088683; doi:10.1186/s12882-021-02351-9)
Supplement: Supplementary file 1 — Additional file 1. [file 12882_2021_2351_MOESM1_ESM.doc]

**SEARCH STRATEGY APPENDIX S1**

**A systematic review and meta-analysis of the omega-3 fatty acids effects on metabolic parameters in dialysis and chronic renal disease patients**

**Javad Heshmati** (corresponding author)

Tehran, Iran.

E-mail: [Javad.Heshmati@gmail.com](mailto:Javad.Heshmati@gmail.com)

Telephone: +98 (021) 44442393

| **Groups** | **Descriptors** |
| --- | --- |
| Outcome | Dialysis OR Hemodialysis OR Peritoneal dialysis OR Kidney disease OR Chronic kidney disease OR End stage renal disease OR Chronic renal failure |
| Exposure | Fish Oil OR Fatty Acids, Omega-3 OR n-3 Fatty Acids OR n-3 Polyunsaturated Fatty Acid OR n-3 PUFA OR alpha Linolenic Acid OR Linolenic Acid OR Linolenate OR “ALA” OR Docosahexaenoic Acids OR “DHA” OR Eicosapentanoic Acid OR “EPA” OR Timnodonic Acid |
| Setting | Randomized controlled trial OR controlled clinical trial OR randomized controlled trials OR random allocation OR double blind method OR single blind method OR clinical trial OR clinical trials OR placebos OR placebo OR random |

**PUBMED**

**Number of localized studies:** 324

**Limits:** humans

**Number of studies after applying limits:** 278

|  | **Descriptors** | Number of studies reached |
| --- | --- | --- |
| **#1** | (((((("renal dialysis"[MeSH Terms] OR ("renal"[All Fields] AND "dialysis"[All Fields]) OR "renal dialysis"[All Fields] OR "dialysis"[All Fields] OR "dialysis"[MeSH Terms]) OR ("haemodialysis"[All Fields] OR "renal dialysis"[MeSH Terms] OR ("renal"[All Fields] AND "dialysis"[All Fields]) OR "renal dialysis"[All Fields] OR "hemodialysis"[All Fields])) OR ("peritoneal dialysis"[MeSH Terms] OR ("peritoneal"[All Fields] AND "dialysis"[All Fields]) OR "peritoneal dialysis"[All Fields])) OR ("kidney diseases"[MeSH Terms] OR ("kidney"[All Fields] AND "diseases"[All Fields]) OR "kidney diseases"[All Fields] OR ("kidney"[All Fields] AND "disease"[All Fields]) OR "kidney disease"[All Fields])) OR ("renal insufficiency, chronic"[MeSH Terms] OR ("renal"[All Fields] AND "insufficiency"[All Fields] AND "chronic"[All Fields]) OR "chronic renal insufficiency"[All Fields] OR ("chronic"[All Fields] AND "kidney"[All Fields] AND "disease"[All Fields]) OR "chronic kidney disease"[All Fields])) OR ("kidney failure, chronic"[MeSH Terms] OR ("kidney"[All Fields] AND "failure"[All Fields] AND "chronic"[All Fields]) OR "chronic kidney failure"[All Fields] OR ("end"[All Fields] AND "stage"[All Fields] AND "renal"[All Fields] AND "disease"[All Fields]) OR "end stage renal disease"[All Fields])) OR ("kidney failure, chronic"[MeSH Terms] OR ("kidney"[All Fields] AND "failure"[All Fields] AND "chronic"[All Fields]) OR "chronic kidney failure"[All Fields] OR ("chronic"[All Fields] AND "renal"[All Fields] AND "failure"[All Fields]) OR "chronic renal failure"[All Fields]) | 742586 |
| **#2** | (((((((((((("Fatty Acids, Omega-3"[Mesh] OR ("fatty acids, omega-3"[MeSH Terms] OR ("fatty"[All Fields] AND "acids"[All Fields] AND "omega-3"[All Fields]) OR "omega-3 fatty acids"[All Fields] OR "n 3 fatty acids"[All Fields])) OR ("fatty acids, omega-3"[MeSH Terms] OR ("fatty"[All Fields] AND "acids"[All Fields] AND "omega-3"[All Fields]) OR "omega-3 fatty acids"[All Fields] OR "n 3 polyunsaturated fatty acid"[All Fields])) OR ("fatty acids, omega-3"[MeSH Terms] OR ("fatty"[All Fields] AND "acids"[All Fields] AND "omega-3"[All Fields]) OR "omega-3 fatty acids"[All Fields] OR "n 3 pufa"[All Fields])) OR ("alpha-linolenic acid"[MeSH Terms] OR ("alpha-linolenic"[All Fields] AND "acid"[All Fields]) OR "alpha-linolenic acid"[All Fields] OR ("alpha"[All Fields] AND "linolenic"[All Fields] AND "acid"[All Fields]) OR "alpha linolenic acid"[All Fields])) OR ("alpha-linolenic acid"[MeSH Terms] OR ("alpha-linolenic"[All Fields] AND "acid"[All Fields]) OR "alpha-linolenic acid"[All Fields] OR ("linolenic"[All Fields] AND "acid"[All Fields]) OR "linolenic acid"[All Fields])) OR ("alpha-linolenic acid"[MeSH Terms] OR ("alpha-linolenic"[All Fields] AND "acid"[All Fields]) OR "alpha-linolenic acid"[All Fields] OR "linolenate"[All Fields])) OR ALA[All Fields]) OR ("docosahexaenoic acids"[MeSH Terms] OR ("docosahexaenoic"[All Fields] AND "acids"[All Fields]) OR "docosahexaenoic acids"[All Fields])) OR DHA[All Fields]) OR ("eicosapentaenoic acid"[MeSH Terms] OR ("eicosapentaenoic"[All Fields] AND "acid"[All Fields]) OR "eicosapentaenoic acid"[All Fields] OR ("eicosapentanoic"[All Fields] AND "acid"[All Fields]) OR "eicosapentanoic acid"[All Fields])) OR EPA[All Fields]) OR ("eicosapentaenoic acid"[MeSH Terms] OR ("eicosapentaenoic"[All Fields] AND "acid"[All Fields]) OR "eicosapentaenoic acid"[All Fields] OR ("timnodonic"[All Fields] AND "acid"[All Fields]) OR "timnodonic acid"[All Fields])) OR "Fish Oils"[Mesh] | 112620 |
| **#3** | ((((((((("Randomized Controlled Trial"[Publication Type] OR "Controlled Clinical Trial"[Publication Type]) OR "Randomized Controlled Trials as Topic"[Mesh]) OR "Random Allocation"[Mesh]) OR "Double-Blind Method"[Mesh]) OR "Single-Blind Method"[Mesh]) OR "Clinical Trial"[Publication Type]) OR ("clinical trial"[Publication Type] OR "clinical trials as topic"[MeSH Terms] OR "clinical trials"[All Fields])) OR "Placebos"[Mesh]) OR ("placebos"[MeSH Terms] OR "placebos"[All Fields] OR "placebo"[All Fields])) OR ("random allocation"[MeSH Terms] OR ("random"[All Fields] AND "allocation"[All Fields]) OR "random allocation"[All Fields] OR "random"[All Fields]) | 1656257 |
| **#4** | **#1** AND **#2** AND #3 | 324 |

**WEB OF SCIENCE**

**Number of localized studies:** 189

**Limits:** documents types (articles)

**Number of studies after applying limits:** 138

|  | **Descriptors** | Number of studies reached |
| --- | --- | --- |
| **#1** | TS=(“Dialysis”) OR TS=(“Hemodialysis”) OR TS=(“Peritoneal dialysis”) OR TS=(“Kidney disease”) OR TS=(“Chronic kidney disease”) OR TS=(“End stage renal disease”) OR TS=(“Chronic renal failure”) | 259639 |
| **#2** | TS=(“Fish oil”) OR TS=(“Fatty Acids, Omega-3”) OR TS=(“n-3 Fatty Acids”) OR TS=(“n-3 Polyunsaturated Fatty Acid”) OR TS=(“n-3 PUFA”) OR TS=(“alpha Linolenic Acid”) OR TS=(“Linolenic Acid”) OR TS=(“Linolenate”) OR TS=(“ALA”) OR TS=(“Docosahexaenoic Acids”) OR TS=(“DHA”) OR TS=(“Eicosapentanoic Acid”) OR TS=(“EPA”) OR TS=(“Timnodonic Acid”) | 116110 |
| **#3** | TS=(Randomized controlled trial) OR TS=(controlled clinical trial) OR TS=(randomized controlled trials) OR TS=(random allocation) OR TS=(double blind method) OR TS=(single blind method) OR TS=(clinical trial) OR TS=(clinical trials) OR TS=(placebos) OR TS=(placebo) OR TS=(random) | 1589270 |
| **#4** | **#1** AND **#2** AND **#3** | 189 |

**SCOPUS**

**Number of localized studies:** 912

**Limits:** *document type* (article and article in press)

**Number of studies after applying limits:** 438

|  | **Descriptors** | Number of studies reached |
| --- | --- | --- |
| **#1** | ( TITLE-ABS-KEY ( dialysis ) ) OR ( TITLE-ABS-KEY ( hemodialysis ) ) OR ( TITLE-ABS-KEY ( peritoneal AND dialysis ) ) OR ( TITLE-ABS-KEY ( kidney AND disease ) ) OR ( TITLE-ABS-KEY ( chronic AND kidney AND disease ) ) OR ( TITLE-ABS-KEY ( end AND stage AND renal AND disease ) ) OR ( TITLE-ABS-KEY ( chronic AND renal AND failure ) ) | 743551 |
| **#2** | ( ( TITLE-ABS-KEY ( fish AND oil ) ) OR ( TITLE-ABS-KEY ( fatty AND acids, AND omega-3 ) ) OR ( TITLE-ABS-KEY ( n-3 AND fatty AND acids ) ) OR ( TITLE-ABS-KEY ( n-3 AND polyunsaturated AND fatty AND acid ) ) OR ( TITLE-ABS-KEY ( n-3 AND pufa ) ) OR ( TITLE-ABS-KEY ( alpha AND linolenic AND acid ) ) OR ( TITLE-ABS-KEY ( linolenic AND acid ) ) OR ( TITLE-ABS-KEY ( linolenate ) ) OR ( TITLE-ABS-KEY ( ala ) ) ) OR ( ( TITLE-ABS-KEY ( docosahexaenoic AND acids ) ) OR ( TITLE-ABS-KEY ( dha ) ) OR ( TITLE-ABS-KEY ( eicosapentanoic AND acid ) ) OR ( TITLE-ABS-KEY ( epa ) ) OR ( TITLE-ABS-KEY ( timnodonic AND acid ) ) ) | 213105 |
| **#3** | ( TITLE-ABS-KEY ( randomized AND controlled AND trial ) OR TITLE-ABS-KEY ( controlled AND clinical AND trial ) OR TITLE-ABS-KEY ( randomized AND controlled AND trials ) OR TITLE-ABS-KEY ( random AND allocation ) OR TITLE-ABS-KEY ( double AND blind AND method ) OR TITLE-ABS-KEY ( single AND blind AND method ) OR TITLE-ABS-KEY ( clinical AND trial ) OR TITLE-ABS-KEY ( clinical AND trials ) OR TITLE-ABS-KEY ( placebos ) OR TITLE-ABS-KEY ( placebo ) OR TITLE-ABS-KEY ( random ) ) | 3087838 |
| **#4** | **#1** AND **#2** AND **#3** | 912 |

**COCHRANE**

**Number of localized studies:** 124

**Limits:** -

**Number of studies after applying limits:** 124

|  | **Descriptors** | Number of studies reached |
| --- | --- | --- |
| **#1** | Me ("Dialysis") or ("Hemodialysis"):ti,ab,kw or ("Peritoneal dialysis"):ti,ab,kw or ("Kidney disease"):ti,ab,kw or ("Chronic kidney disease"):ti,ab,kw or ("End stage renal disease"):ti,ab,kw or ("Chronic renal failure"):ti,ab,kw | 23680 |
| **#2** | Me ("Fish Oil") or ("Fatty Acids, Omega-3"):ti,ab,kw or ("n-3 Fatty Acids"):ti,ab,kw or ("n-3 Polyunsaturated Fatty Acid"):ti,ab,kw or ("n-3 PUFA"):ti,ab,kw or ("alpha Linolenic Acid"):ti,ab,kw or ("Linolenic Acid"):ti,ab,kw or ("Linolenate"):ti,ab,kw or ("ALA"):ti,ab,kw or ("Docosahexaenoic Acids"):ti,ab,kw or ("DHA"):ti,ab,kw or ("Eicosapentanoic Acid"):ti,ab,kw or ("EPA"):ti,ab,kw or ("Timnodonic Acid"):ti,ab,kw | 5887 |
| **#3** | **#1** AND **#2** | 124 |

**EMBASE**

**Number of localized studies:** 922

**Limits:** *publication type* (article)

**Number of studies after applying limits:** 427

|  | **Descriptors** | Number of studies reached |
| --- | --- | --- |
| **#1** | 'dialysis'/exp OR dialysis OR 'hemodialysis'/exp OR hemodialysis OR (peritoneal AND ('dialysis'/exp OR dialysis)) OR (('kidney'/exp OR kidney) AND ('disease'/exp OR disease)) OR (chronic AND ('kidney'/exp OR kidney) AND ('disease'/exp OR disease)) OR (end AND stage AND ('renal'/exp OR renal) AND ('disease'/exp OR disease)) OR (chronic AND ('renal'/exp OR renal) AND ('failure'/exp OR failure)) | 1317236 |
| **#2** | ('fish'/exp OR fish) AND ('oil'/exp OR oil) OR (fatty AND acids, AND ('omega 3'/exp OR 'omega 3')) OR ('n 3' AND fatty AND ('acids'/exp OR acids)) OR ('n 3' AND polyunsaturated AND fatty AND ('acid'/exp OR acid)) OR ('n 3' AND pufa) OR (alpha AND linolenic AND ('acid'/exp OR acid)) OR 'linolenic acid'/exp OR 'linolenic acid' OR 'linolenate'/exp OR linolenate OR ala OR (docosahexaenoic AND ('acids'/exp OR acids)) OR 'dha'/exp OR dha OR (eicosapentanoic AND ('acid'/exp OR acid)) OR epa OR (timnodonic AND ('acid'/exp OR acid)) | 175414 |
| **#3** | (randomized AND controlled AND ('trial'/exp OR trial)) OR (controlled AND ('clinical'/exp OR clinical) AND ('trial'/exp OR trial)) OR (randomized AND controlled AND trials) OR (random AND allocation) OR (double AND ('blind'/exp OR blind) AND ('method'/exp OR method)) OR (single AND ('blind'/exp OR blind) AND ('method'/exp OR method)) OR (('clinical'/exp OR clinical) AND ('trial'/exp OR trial)) OR (('clinical'/exp OR clinical) AND trials) OR ('placebos'/exp OR placebos) OR ('placebo'/exp OR placebo) OR (random) | 2685676 |
| **#4** | **#1** AND **#2** AND **#3** | 922 |
