# Supplementary material for: Effect of omega-3 fatty acids supplementation on cardio-metabolic and oxidative stress parameters in patients with chronic kidney disease: a systematic review and meta-analysis
Source: BMC Nephrol. 2021 May 1;22:160. doi: 10.1186/s12882-021-02351-9 (PMC8088683; doi:10.1186/s12882-021-02351-9)
Supplement: Supplementary file 2 — Additional file 2. [file 12882_2021_2351_MOESM2_ESM.docx]

***Appendix 2: Assessment of the risk of bias in the included studies***

| **Author, year** | **Random sequence generation (selection bias)** | **Allocation concealment (selection bias)** | **Blinding of participants and personnel (performance bias)** | **Blinding of outcome assessment (detection bias)** | **Incomplete outcome data (attrition bias)** | **Selective reporting (reporting bias)** | **Other bias** |
| --- | --- | --- | --- | --- | --- | --- | --- |
| Ando et al [25] (1999) | **?** | **_** | **+** | **+** | **?** | **+** | **_** |
| Ateya et al [26] (2017) | **?** | **_** | **_** | **?** | **_** | **_** | **_** |
| Bouzidi et al [27] (2010) | **?** | **?** | **?** | **_** | **+** | **_** | **_** |
| Alexopoulos et al [24] ( 2004) | **_** | **?** | **+** | **+** | **?** | **?** | **_** |
| Gharekhani et al [28] (2016) | **_** | **_** | **?** | **+** | **?** | **_** | **_** |
| Jabbari et al [29] (2016) | **_** | **?** | **+** | **+** | **?** | **_** | **_** |
| Khajehdehi [30] (2000) | **?** | **?** | **+** | **+** | **?** | **_** | **_** |
| Moeinzadeh [31] et al (2011) | **_** | **_** | **?** | **?** | **+** | **_** | **_** |
| Naini et al [32] (2015) | **_** | **_** | **_** | **_** | **?** | **_** | **_** |
| Pettersson [33] et al (1994) | **+** | **?** | **_** | **?** | **+** | **+** | **_** |
| Schmitz [34] et al (2002) | **_** | **_** | **_** | **?** | **?** | **_** | **_** |
| Tayyebi-Khosroshahi et al [35] (2013) | **_** | **_** | **?** | **+** | **?** | **?** | **_** |
| Tayyebi-Khosroshahi et al [36] (2010) | **+** | **?** | **+** | **+** | **?** | **_** | **_** |

**+: High risk, -: Low risk, ?:Unclear**
