# Supplementary material for: Effect of omega-3 fatty acids supplementation on cardio-metabolic and oxidative stress parameters in patients with chronic kidney disease: a systematic review and meta-analysis
Source: BMC Nephrol. 2021 May 1;22:160. doi: 10.1186/s12882-021-02351-9 (PMC8088683; doi:10.1186/s12882-021-02351-9)
Supplement: Supplementary file 3 — Additional file 3. [file 12882_2021_2351_MOESM3_ESM.docx]

A

B

C

D

Appendix 3: Funnel plots of standard error by standardized mean differences of TC (A), HDL (B), LDL (C), and TG (D).
